# 

**Published:** 1847-06

**Authors:** 


### CONTENTS

OF NO. VI.

### ORIGINAL COMMUNICATIONS.

### ESSAYS AND CASES.

ART. I.—Notes on Medical Matters and Medical Men in Paris. By DAVID W. YANDELL, M. D., of Louisville, Ky. - - - - - - - - - - 461

ART. II.—A Case of Fistulous Opening of the Lung: Clinical Lectures in New York. By B. F. WENDEL, M. D., of Murfreesboro', Tennessee. - - - - - 497

ART. III.—A Case of Acute Amenorrhea, Pneumonia, Enlargement of the Liver. By JOHN T. MARABLE, M. D., of Montgomery County, Tenn. - - - - - 502

### REVIEWS.

ATR. IV.—A Practical Treatise on the Diseases of Children. By D. FRANCIS CONDIE, M. D., Secretary of the College of Physicians; Honorary Member of the Philadelphia Medical Society, etc. Second edition, revised and augmented. - - - - - - - - - 505

ART. V.—Transactions of the Medical Society of the State of New York, vol. ii, part i. 1847. - - - - - 518

### SELECTIONS FROM AMERICAN AND FOREIGN JOURNALS.

Gun Cotton in Blasting. - - - - - - - - - 521
Structure of the Sea-serpent. - - - - - - - 522
Plagiarism. - - - - - - - - - - - 523

Vapor of Ether in Asthma and Hooping Cough. . - - 524
Advance of Asiatic Cholera. - - - - - - - - 525
Cholera in the Russian Sanatory Cordon. - - - - - 525
Diseases of the Digestive Organs. - - - - - - 525
Rupture of the Umbilical Cord, and of the Vagina. - - 529
Affection of Peyer's Glands in Adynamic Fever. - - - 534
Prof. Chapman on Medical Reform. - - - - - - 534
Gunpowder. - - - - - - - - - - - 535
Diseases of the Army at Monterey. - - - - - - 537

ORIGINAL INTELLIGENCE.

Medical Convention. - - - - - - - - - 540
Death of Prof. Worcester. - - - - - - - - 542
Death of Prof. Revere. - - - - - - - - 543
Death of Dr. George McClellan. - - - - - 543
Prison Discipline. - - - - - - - - - 544
Dr. Harris and the New Orleans Journal of Medicine. - - 546
Ohio Medical Convention. - - - - - - - 547

